# Optimising spatial accessibility to inform rationalisation of specialist health services

**DOI:** 10.1186/s12942-017-0088-6

**Published:** 2017-04-21

**Authors:** Catherine M. Smith, Hannah Fry, Charlotte Anderson, Helen Maguire, Andrew C. Hayward

**Affiliations:** 1 UCL Department of Infectious Disease Informatics, Farr Institute of Health Informatics Research, University College London, London, UK; 2 Centre for Advanced Spatial Analysis, University College London, London, UK; 3 Field Epidemiology Service – South East and London, Public Health England, London, UK; 4 Research Department Infection and Population Health, Centre for Infectious Disease Epidemiology, University College London, London, UK

**Keywords:** Spatial accessibility, Optimisation, Travel time, Service planning, Specialist services

## Abstract

**Background:**

In an era of budget constraints for healthcare services, strategies for provision of services that improve quality whilst saving costs are highly valued. A proposed means to achieve this is consolidation of services into fewer specialist centres, but this may lead to reduced spatial accessibility. We describe a methodology which includes implementing a combinatorial optimisation algorithm to derive combinations of services which optimise spatial accessibility in the context of service rationalisation, and demonstrate its use through the exemplar of tuberculosis clinics in London.

**Methods:**

Our methodology involves (1) identifying the spatial distribution of the patient population using the service; (2) calculating patient travel times to each service location, and (3) using a combinatorial optimisation algorithm to identify subsets of locations that minimise overall travel time. We estimated travel times for tuberculosis patients notified in London between 2010 and 2013 to each of 29 clinics in the city. Travel time estimates were derived from the Transport for London Journey Planner service. We identified the subset of clinics that would provide the shortest overall travel time for each possible number of clinic subsets (1–28).

**Results:**

Based on the 29 existing clinic locations, mean estimated travel time to clinics used by 12,061 tuberculosis patients in London was 33 min; and mean time to their nearest clinics was 28 min. Using optimum combinations of clinic locations, and assuming that patients attended their nearest clinics, a mean travel time of less than 45 min could be achieved with three clinics; of 34 min with ten clinics, and of less than 30 min with 18 clinics.

**Conclusions:**

We have developed a methodological approach to optimise spatial accessibility which can be used to inform rationalisation of health services. In urban conurbations, this may enable service reorganisation which increases quality and efficiency without substantially affecting spatial accessibility. This approach could be used to inform planning of service reorganisations, but may not be generalisable to rural areas or smaller urban centres.

## Background

In an era of budget constraints for healthcare services, strategies for provision of services that improve quality whilst saving costs are highly valued. For specialist services, such as major trauma, stroke care and cardiac surgery, a proposed means to achieve this is consolidation of service provider locations into fewer centres [1–3]. Potential benefits of concentration of care include increased levels of expertise; reduced variation in quality, and simplification of care networks. In London, for example, recent reconfiguration of stroke services involved reducing the number of hospitals providing acute stroke care from 30 to 8 [4]. This was associated with significant decreases in stroke-associated mortality and length of hospital stay [4].

A potential drawback of providing care from a smaller number of locations is that it may reduce accessibility to services. Healthcare accessibility is a multi-dimensional concept that is influenced by spatial and aspatial factors [5]. It has been defined as comprising five dimensions: availability, accessibility, affordability, acceptability and accommodation [6]. Availability and accessibility are inherently spatial factors describing, respectively, the number of services in comparison to the number of potential users of services, and the burden of travel between locations [7]. The latter three dimensions, conversely, reflect financial and cultural attitudes and are therefore largely aspatial [8].

Ensuring adequate spatial accessibility for healthcare services requires knowledge of the spatial distribution of the patient population. For specialist services, this may vary considerably from the distribution of the general population and should therefore be taken into account when planning services. Spatial accessibility can be considered a measure of the friction or cost of travelling between locations [7]. It can be quantified in various ways including Euclidian (straight line) or network (along a path) distances, and travel time. In cities with extensive public transport networks, travel time offers a more accurate representation of the cost of travel [7]. It is also becoming increasingly available, with online services providing estimates of journey times using different modes of transport.

**Fig. 1:**
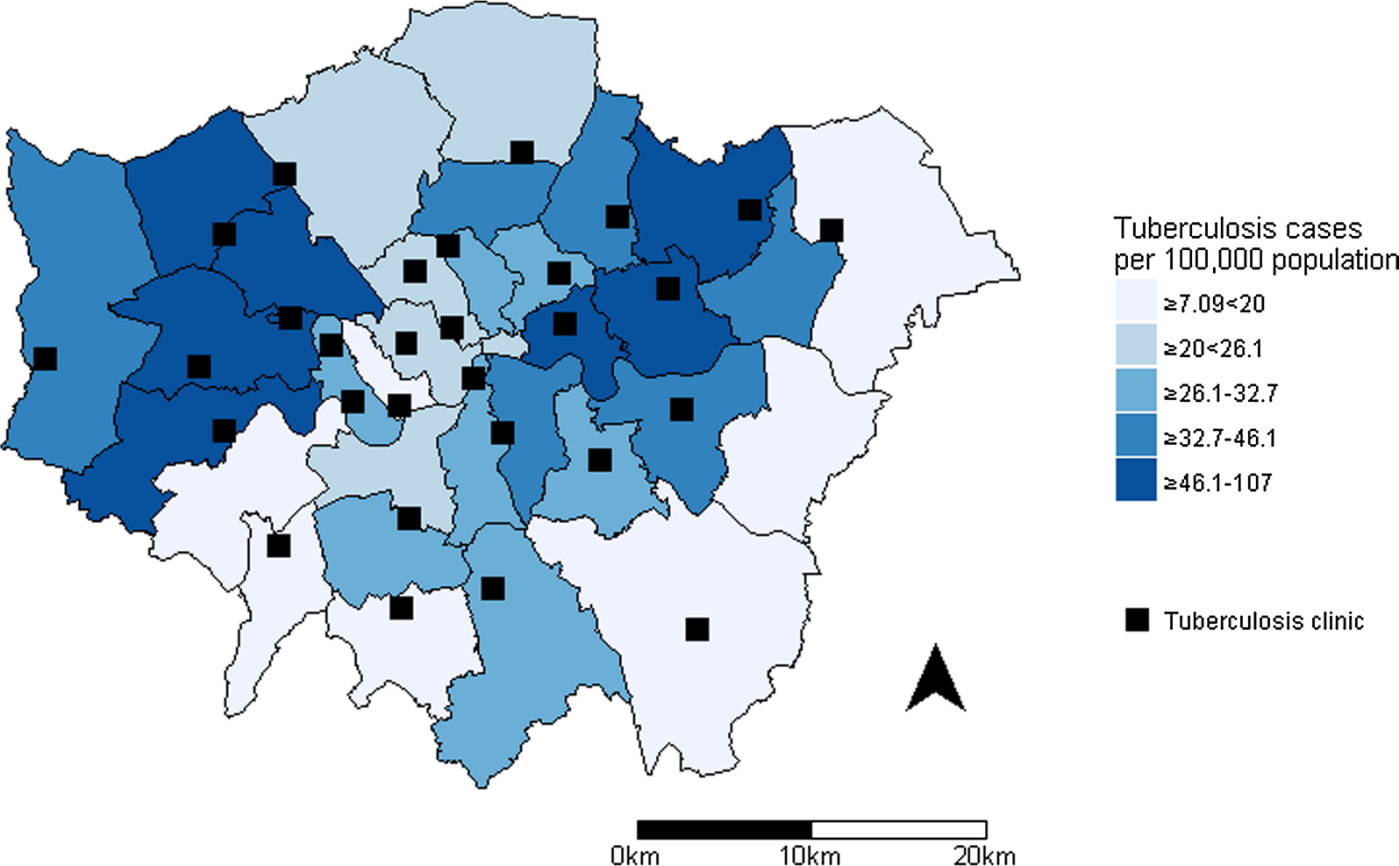
Average annual incidence of tuberculosis in London by local authority, 2010–2013, with locations of 29 tuberculosis clinics. Contains Ordnance Survey data © Crown copyright and database right 2014

In this study, we propose a methodology based on spatial accessibility for selecting optimum combinations of service locations in the context of service rationalisation in metropolitan areas. We have chosen to demonstrate this method through the exemplar of tuberculosis services in London for several reasons: First, tuberculosis is a highly geographically dependent disease due to its association with ethnicity and deprivation (Fig. 1) [9]. Rates of disease and service requirements therefore vary in different parts of the city. Second, precise geographic information is available for all patients through the Public Health England Enhanced Tuberculosis Surveillance (ETS) system.

Third, spatial accessibility is a particularly important consideration for tuberculosis patients because of their long treatment duration (6 months standard regimen) requiring multiple journeys to the clinic. Patients on directly observed therapy have to travel to clinics three to five times per week, which is burdensome and potentially stigmatising. Reducing travel time for these patients could therefore have implications for treatment completion rates as well as lowering economic costs for patients [10].

Finally, there are currently more than 30 tuberculosis clinics in London, to which patients are assigned based on arrangements with the 32 Clinical Commissioning Groups (CCGs) covering the region. However, it has recently been suggested that tuberculosis services may benefit from being commissioned at the city level [11, 12]. This would present an opportunity for rationalisation of tuberculosis services in London, which are provided at fewer clinics in other comparable cities such as Paris, Barcelona and New York [11, 13, 14]. This has potential to benefit patient outcomes as there is some evidence that smaller caseloads, which arise from distributed services, are associated with failure to complete tuberculosis treatment [15].

The aim of this study was to develop and describe a methodology to derive combinations of service locations which optimise spatial accessibility in the context of service rationalisation, and to demonstrate its use through the exemplar of tuberculosis clinics in London.

## Methods

The methodology that we have developed involves (1) identifying the spatial distribution of the patient population using the service; (2) calculating patient travel time to each service location, and (3) using a combinatorial optimisation algorithm to identify sets of locations that minimise overall travel time.

### Data sources and travel time data

We applied this method to clinic travel times for cases of tuberculosis who resided in London and were notified between 1 January 2010 and 31 December 2013. We extracted the residential post codes and clinic that was attended by each patient from the ETS system. We excluded small or specialist clinics and the patients who attended them, because we aimed to generate a realistic assessment of the accessibility of clinics that were available for all patients to attend. Exclusions included children aged under 18 years (who were eligible to attend a clinic at a specialist children’s hospital), and three clinics that had served fewer than 30 patients over the 4 years of the study. A total of 29 clinics and the patients attending them were therefore included. We additionally excluded patients whose post code was the same as that of the clinic, because this post code is used when the residential address of the patient is unknown.

Estimates of patient travel times were derived from the Transport for London (TfL) Journey Planner service (https://tfl.gov.uk/plan-a-journey). This is an online application which allows users to calculate approximate travel times between any two locations served by the London public transport system. The service can also be accessed through an application programming interface (API), which allows the Journey Planner to be queried programmatically through HTTP requests. We used the Journey Planner service, accessed through its API using the R package XML [16], to estimate the minimum travel time from each patient residential location to each tuberculosis clinic in London. We set the journeys at an arbitrary week day date and time outside of usual rush hour services (10:30 a.m.), under the assumption that the majority of clinic appointments would be attended at these times.

### Comparison of used and catchment clinics

We defined the clinic that the patient attended as their ‘used’ clinic; and the clinic which would provide the shortest estimated travel time as their ‘catchment’ clinic (i.e. the one for which they were in the catchment area based on travel times).

We calculated the overall mean patient travel time to the used and catchment clinics and compared them using a *t* test. We plotted the distribution of travel times for patients’ used and catchment clinics and summarised the difference in number of patients within different travel time thresholds (15, 30 and 60 min). We also determined the impact on clinic caseloads if all patients attended their catchment clinic.

### Optimum clinic configurations to minimise travel time

We investigated the optimal theoretical combinations of subsets of clinic locations using a combinatorial optimisation algorithm. The aim of this analysis was to determine, for each set of n clinics in London, which group of n clinics would provide minimum overall patient travel time. For example, if there were to be seven clinics in the city, which group of seven of the 29 current clinics would minimise overall patient travel time. Theoretically, this could be determined by calculating the total travel time for all possible combinations of seven clinics. However, this is not computationally feasible in practice, because the number of combinations of clinics becomes very large with increasing numbers of clinics being selected. This can be calculated as follows.

The number of combinations of *n* distinct clinics, taken *r* at a time is:

$${\text{nCr }} = {\text{n}}!/ {\text{r}}! \left( {{\text{n}} - {\text{r}}} \right)!$$

For example, choosing from 29 clinics in groups of seven:

$$\begin{aligned} & {\text{n}} = 29; {\text{r}} = 7 \\ & 29{\text{C}}7 = 29!/ 7!\left( {29 - 7} \right)! = 1{,}560{,}780 \\ \end{aligned}$$

Therefore, to test all possible combinations of seven clinics, minimum travel times would have to be determined for each patient for more than 1.5 million different sets.

Optimisation algorithms are designed to solve problems such as these in which an exhaustive search is not feasible. A combinatorial optimisation algorithm was required in this case because it uses discrete variables that can represent quantities that can only be integers, such as clinics. This is distinct from a continuous optimisation problem in which the solution represents, for example, the mass of an object, and may take any value [17]. We used the algorithm provided by the rgenoud package in R [18]. It has been applied for optimisation of parameters in various different fields including healthcare evaluation [19], ecological modelling [20], and animal behaviour modelling [21].

The rgenoud package (described in detail elsewhere [18, 22]) employs a genetic evolutionary algorithm. Genetic algorithms are a class of combinatorial optimisation algorithm which are inspired by the processes of evolutionary genetics. The algorithm begins the search for the optimum result with a random sample of candidate solutions, termed a ‘population’. The population is then ‘evolved’ through multiple generations towards better solutions, using logical operations based on the evolutionary processes of mutation, crossover and selection [22]. With each generation, the average ‘fitness’ (the closeness to the solution) of the population generally increases, and the process is repeated through multiple generations until a termination condition is reached. Therefore in this application of the algorithm, the optimal solution is the combination of clinics that has the minimum total patient travel time. The first population is a random selection of different combinations of clinics, and the combinations with the higher fitness (shortest travel time) are selected to create the next generation. Mutation and crossover introduce random changes to the combinations of clinics in the next generation. The process is repeated until several generations pass without any combinations with shorter overall travel times being produced.

To identify an optimum set of clinics based on minimum patient travel time, we used travel times for a random sample of 1000 patients (8.3% total). The random sample was used to limit computation time. We derived optimum combinations of clinics for each possible total number of clinics (subsets of one to 28 of the 29 total clinics). We determined the impact on travel time for all patients, i.e. including those not in the random sample on which the optimisation algorithm was run. We calculated the caseload for clinics based on the catchments when using optimum sets.

## Results

**Fig. 2:**
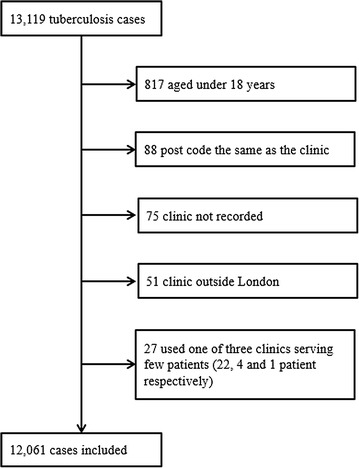
Cases included in analysis of tuberculosis service accessibility in London, 2010–2013

There were 13,119 cases of tuberculosis in patients notified with tuberculosis between 2010 and 2013 who resided in London. Of these, 817 were excluded from the analysis because they were aged under 18 years (eligible to attend a clinic at a specialist children’s hospital); 88 were excluded because their post code was recorded as the same as the clinic (used when the residential address of the patient is unknown); 75 because their clinic was not recorded; 51 because they used a clinic outside London, and 27 because they used one of three tuberculosis clinics that serve fewer than 30 patients (Fig. 2). A total of 12,061 patients with viable post codes attending the 29 clinics in London were therefore included.

### Comparison of used and catchment clinics

**Fig. 3:**
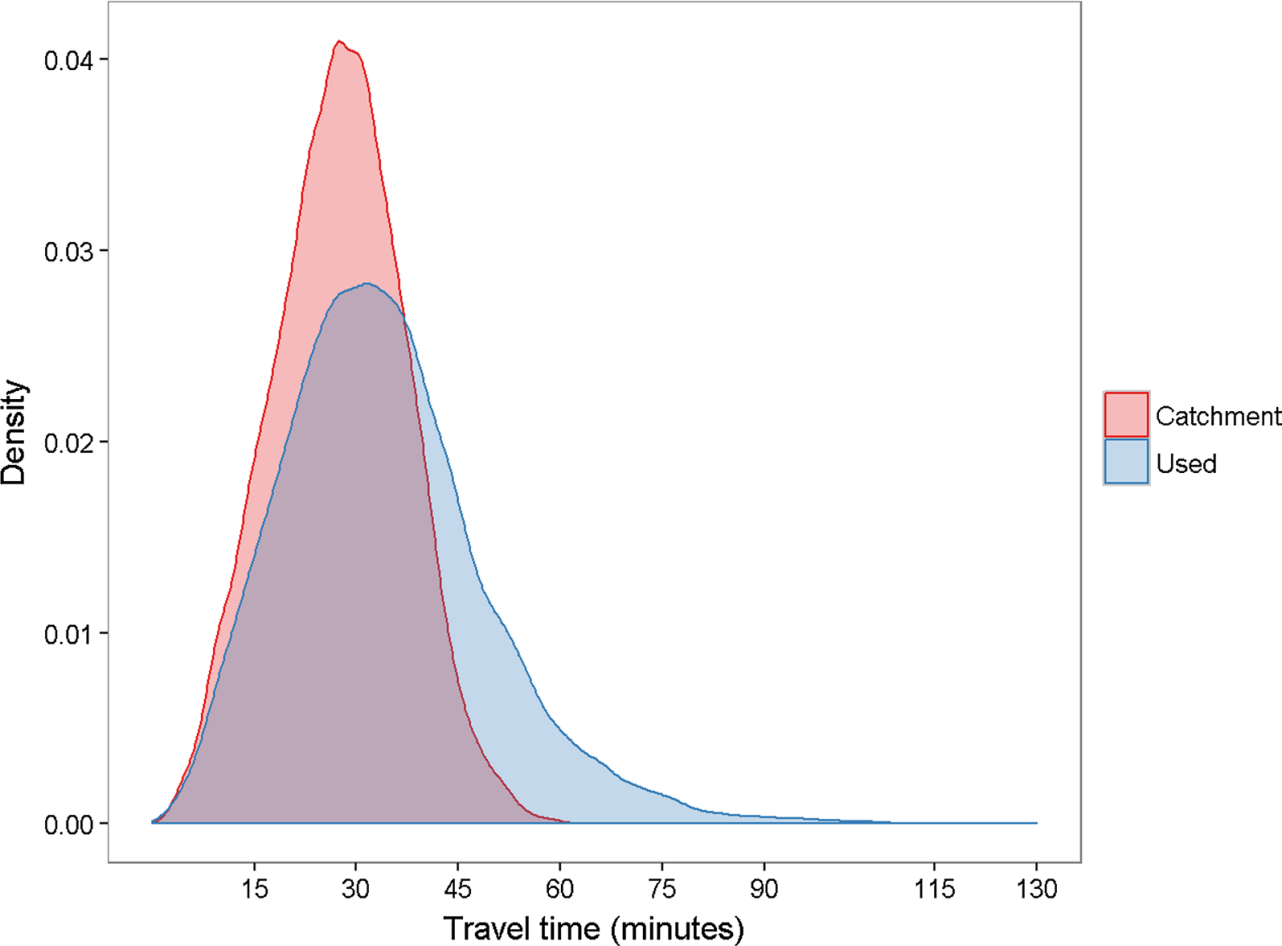
Distribution of estimated travel times for tuberculosis patients to used and catchment clinics, London, 2010–2013. N = 12,061 patients; catchment clinic defined for each patient as the clinic that would provide the shortest travel time. Density function generated using standard Gaussian kernel density estimator

Figure 3 shows the distribution of estimated travel times for used and catchment clinics. The mean travel time to used clinics was 33 min (standard deviation 15.1 min). Catchment clinics would provide a small but significant decrease in average travel times (mean 27.5 min, standard deviation 9.6 min, *t* test p < 0.01). A total of 7337 (61%) patients used their catchment clinic; 2130 (18%) used a clinic more than 15 min further than their nearest clinic; 767 (6%) more than 30 min, and 59 (0.5%) more than 60 min.

The median number of patients using each clinic over the 4 years of the study was 369 (IQR 252–416), and in clinic catchments was 400 (IQR 205–510). There were 12 clinics that served more patients than in their catchment, 16 served fewer than in their catchment, and one served the same number. The clinic with the largest change in caseload would have served 368 more patients if it included all those in its catchment, a 35% increase.

**Fig. 4:**
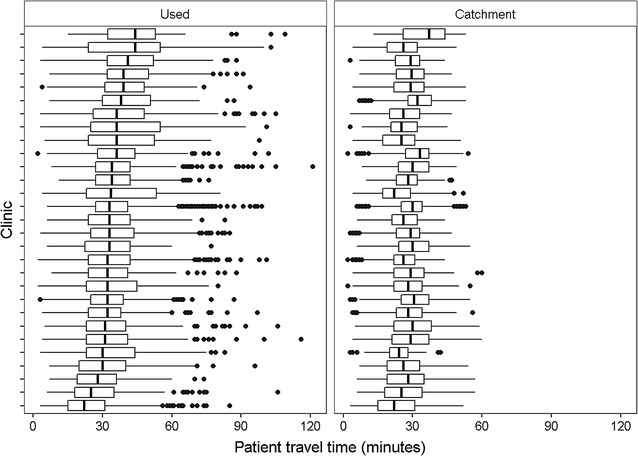
Distribution of tuberculosis patient estimated travel times to 29 clinics in London, 2010–2013. N = 12,061 patients; catchment clinic defined for each patient as the clinic that would provide the shortest travel time. Each *box plot* shows the distribution of travel times for a different clinic, with *vertical lines* representing lower quartiles, median, and upper quartiles, and *dots* representing outliers

Box plots of the numbers of patients by clinic show that assigning patients by catchment produces a smaller range in travel times by clinic, and patient travel times would be more consistently under 30 min (Fig. 4).

### Optimum clinic configurations to minimise travel time

**Fig. 5:**
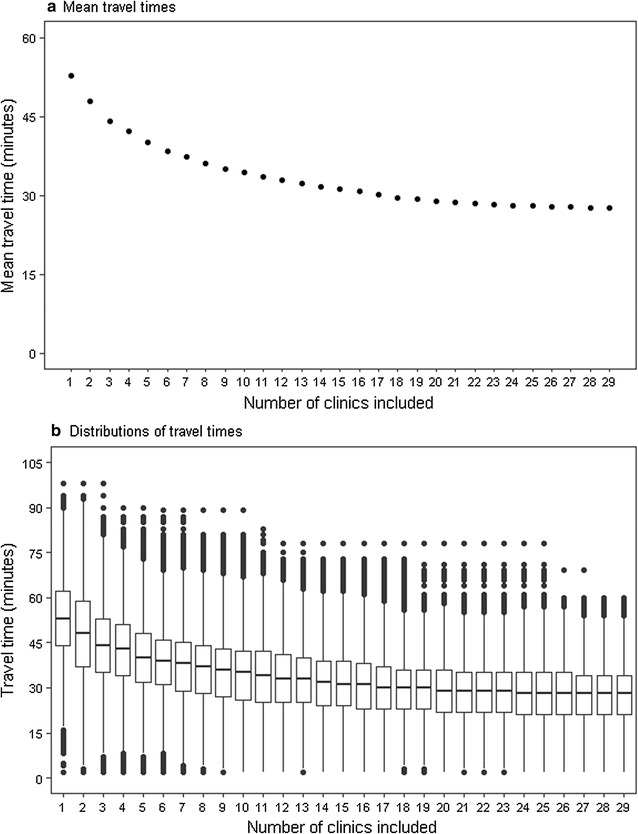
Estimated patient travel times to tuberculosis clinics for optimum clinic configurations, London, 2010–2013. Patients assigned to clinics based on minimum travel times. **a** *Dots* represent mean travel times for each number of included clinics. **b** *Box plots* show distribution of travel times for each number of clinics included, with *horizontal lines* representing lower quartiles, median, and upper quartiles, and *dots* representing outliers

The optimum configurations of clinics to minimise total patient travel time were determined using the combinatorial optimisation algorithm. Average patient travel times and distributions of travel times for each set of n clinics are shown in Fig. 5a, b. As would be expected, if only one clinic was used, its optimum location is the centre of the city, but the average travel times are long (53 min). Adding further clinics to this one has a substantial impact on average travel time up to approximately ten clinics (mean travel time 34 min), after which the benefits in accessibility become more marginal with additional clinics (mean travel time for 29 clinics 27.5 min). Maximum travel times suffer more from including only ten clinics (89 min) compared to all 29 clinics (60 min) (Fig. 5b); however the travel time for the 95th percentile patient would be only 10 min longer (53 min) when using ten clinics compared to all 29 clinics (43 min). Using the optimum combination of three clinics, and assuming that patients used their catchment clinic, mean travel time would be less than 45 min; with the optimum combination of 18 clinics, mean travel time would be less than 30 min.

**Fig. 6:**
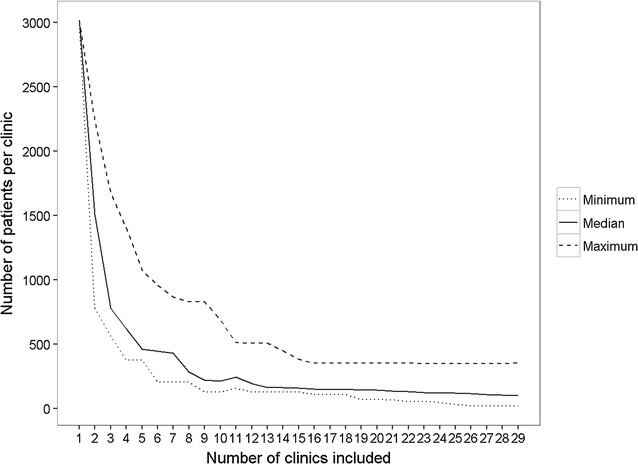
Average annual number of tuberculosis patients per clinic for optimum clinic configurations, London, 2010–2013. Patients assigned to clinics based on minimum travel times

Figure 6 summarises the average annual number of patients that would have attended each clinic for each number of clinics included in the optimum set. The graph shows a similar pattern to the change in travel times with increased numbers of clinics included. A sharp decline in the number of patients per clinic is evident with addition of clinics, up to approximately ten clinics (median 855 patients, IQR 715–1571).

## Discussion

In this study, we have demonstrated a methodology for rationalisation of specialist healthcare services in a major urban conurbation which minimises impacts on spatial accessibility. It involves deriving patient travel time to all service locations, and using a combinatorial optimisation algorithm to determine sets of locations that result in shortest overall patient travel time.

In the context of tuberculosis services in London, we have shown that current mean patient travel times could be achieved with an optimum combination of around ten of the 29 clinic locations in London, provided that patients were able to attend their nearest clinic. An advantage of the approach that we have developed is that it can be used to assess impacts on spatial accessibility when including different numbers of service locations. It could therefore be used to inform evidence-based decisions when planning service rationalisation, both about the number and location of services required to meet acceptable travel time thresholds.

To our knowledge, this is the first time that a study of service accessibility has used a combinatorial optimisation approach to identify optimum service configurations. Previous studies of service accessibility include measures of provider-population relationships, such as simple ratios of provider supply to patient demand [23]; and gravity models, which account for the diminishing attractiveness of services with increased distance [24]. A more sophisticated technique is the two-step floating catchment area method, which brings together elements of provider-population relationship and gravity models [5]. It consists of overlapping catchments of both service provision and resident utilisation, and can therefore measure availability relative to demand and distance between services and residents. However, these methods were designed to identify areas with poor or good accessibility, rather than to inform service rationalisation.

The methodology that we have described has broad potential applicability as reconfiguration of specialist services into smaller numbers of centres is increasingly becoming part of health service planning. In Denmark, for example, there is an ongoing programme of specialisation and centralisation of care resulting in establishment of five ‘super hospitals’ by 2020 [25]. This includes reform of stroke care, which is proposed to be concentrated at two centres, selected based on patient volumes. Stroke care has also been reconfigured in two major cities in England, London and Greater Manchester [26]. In London, hospital selection involved identification of potential sites based on determination of need and including ambulance travel times [4]. We envisage the approach outlined in this study being used to inform planning stages of similar future service reorganisations. It provides a formal, transparent methodology which would also be useful, for example, for communication of decisions to the public. In areas with less extensive public transport systems, travel time could be derived from network distances or driving times. However, we acknowledge that the methods may not be generalisable to rural areas or smaller urban centres.

An important limitation of this method is that it does not take into account other dimensions of healthcare accessibility that are important when planning services. For example, clinic facilities, space and staffing would influence whether it could accommodate additional patients. When implementing this method in practice, it would therefore be important to synthesise results with measures of these aspects of accessibility, as well as exploring patient preferences and consulting with health professionals.

The example of tuberculosis clinic accessibility in this study was based on a number of assumptions, primarily that the analysis used estimated rather than actual travel times. By selecting the minimum travel time from residential locations to clinics, we assumed that patients would always have a preference for the shortest route in time. In reality, there are other factors that contribute to the choice of journey, such as the number of changes between modes of transport and the price of the journey. Furthermore, patients may opt to travel to the clinic from a location other than their home, for example from their workplace. These issues could alter the realised accessibility of the clinics. There are also other considerations when planning locations of tuberculosis services, including the cost of service reorganisations and provision of local care in closer community settings. ‘Hub and spoke’ models, in which care is coordinated at a smaller number of centres and additional services such as staff home visits and contact tracing are provided by satellite units or other existing services such as pharmacies and district nurses, may be a way of applying the methods described here whilst maintaining local links [27].

Another assumption of the study was that the TfL Journey Planner provides an accurate estimate of the travel time between two locations. Walking speeds, for example, will vary between individuals and may be longer for people who are suffering with tuberculosis than the general population. We may therefore have underestimated travel times for which a large proportion of the journey would be made on foot. Travel times may also be affected by the time of day, for example due to the number of services available and how busy they are. We set the journey time at 10.30 a.m. on a week day, which may have underestimated travel time when compared to more typically busy periods.

The combinatorial optimisation approach provides an objective means of assessing optimal combinations of clinics which is cheap and quick to calculate. A limitation of this analysis is that, since the algorithm does not test every combination, it cannot be guaranteed to have found the optimum. For example, the algorithm may converge on local optima rather than the global optimum solution [22]. However in this study, we re-ran the optimisation algorithm for each possible total number of clinics (from 1 to 28). For each increasing number of clinics, the optimum combination identified included the subset of clinics in the previous combination, as opposed to a new subset, although this was not set as an initial condition of the algorithm. It is therefore unlikely that the algorithm had become ‘stuck’ in local optima as it would have had to occur in the same way on multiple occasions.

In future, this methodology could inform arrangement of other services, public and commercial, in addition to wider applicability to health service reorganisations. A similar approach could also be used to consider different alternative combinations of new locations of service given a set of potential options. For example, specialist treatment centres for management of multidrug-resistant tuberculosis in England have recently been established. These centres had to fulfil certain criteria including a minimum annual case load of tuberculosis patients. Use of the methodology described here could have informed this decision by also considering the spatial accessibility of locations for the relevant population [28].

## Conclusions

We have developed a methodological approach to optimise spatial accessibility which can be used to inform rationalisation of health services. In urban conurbations, this may allow increased efficiency and quality of specialist services without substantially affecting spatial accessibility. This approach could be used to inform planning of service reorganisations, but may not be generalisable to rural areas or smaller urban centres.

## Abbreviations

API: application programming interface
CCG: Clinical Commissioning Group
ETS: enhanced tuberculosis surveillance
TfL: Transport for London
